# A dataset examining technical factors on fixed white blood cell single-cell RNA-seq

**DOI:** 10.1016/j.dib.2024.111096

**Published:** 2024-10-31

**Authors:** Daniel V Brown, Agnieszka Swierczak, Yue You, Yupei You, Daniela Amann-Zalcenstein, Peter Hickey, Arthur Hsu, Matthew E. Ritchie, Monther Alhamdoosh, Judith Field, Rory Bowden

**Affiliations:** a The Walter and Eliza Hall Institute of Medical Research, Parkville, Victoria 3052, Australia; b CSL Innovation, Bio21 Institute, Parkville VIC, Australia; c Guangzhou National Laboratory, Guangzhou, China

**Keywords:** 10x genomics flex, Preservation, Neutrophils, Transcriptome

## Abstract

Single-cell RNA sequencing (scRNA-seq) is a powerful technology that enables the measurement of gene expression in individual cells. Such precision provides insights into cellular heterogeneity that bulk methods might overlook. Fragile cells, in particular neutrophils, have posed significant challenges for scRNA-Seq due to their *ex vivo* fragility, high RNase content and consequent loss during cryopreservation. The introduction of fixed scRNA-Seq methodology offers a promising solution to these challenges. We evaluated the performance of two different commercial platforms on red blood cell-depleted whole blood cells: 10x Genomics Flex v1 and Honeycomb HIVE v1.

These data are publicly available from the Gene Expression Omnibus database (accession number GSE266615).

Further insights could be gained by correcting batch and technical effects introduced by storage time after fixation and cell numbers fixed. These data may be used to examine how reflective the transcriptome of neutrophils are of the native environment.

Specifications Table

**Table:** 

Subject	Biological sciences, Omics: Transcriptomics
Specific subject area	Single-cell transcriptomics on red blood cell-depleted, fixed whole blood
Data format	Fastq (.fastq) and R Data Serialization (.rds) object
Type of data	Raw fastq and pre-processed cell by gene count matrix
Data collection	Blood samples from healthy volunteers were collected into either NaCit or EDTA collection tubes. RBCs were lysed in ACK buffer. Fixation was performed according to each manufacturer-specific protocol. Honeycomb HIVE libraries were processed with cat 10347 v.0.3 revA. Batch 1 and 2 experiments were processed with Chromium Fixed RNA Profiling CG000527 Rev. B. Samples were sequenced on an Illumina NextSeq2000. HIVE libraries were pre-processed with Honeycomb BeeNet, v1.1. 10x Genomics Flex libraries were pre-processed with Cell Ranger multi v7.1.0.
Data source location	• *Institution: Walter and Eliza Hall Institute* • *City/Town/Region: Parkville, Victoria* • *Country: Australia*
Data accessibility	Repository name: Gene Expression Omnibus (GEO) Data identification number: GSE266615 Direct URL to data: https://www.ncbi.nlm.nih.gov/geo/query/acc.cgi?acc=GSE266615 Instructions for accessing these data: Preprocessing and analysis scripts of these data are available from https://github.com/WEHIGenomicsRnD/DiB_fixed_WBC_scRNAseq

## 1 Value of the Data

• These data are useful as they enable measurement of the effect of fixation on single-cell transcriptomes from white blood cells.
• These data could be used to examine technical factors that affect data quality, especially cell number inputs.
• Data could also be used to assess the performance of automated cell annotation methods on fixed scRNA-Seq data.
• Further insights can be gained by correcting for batch and technical effects and subsequently examining the underlying biology.

## 2 Background

High-throughput sequencing technologies have enabled many biological discoveries; nevertheless, the resolution of bulk RNA sequencing is insufficient to elucidate heterogeneity at the single-cell level. Single-cell RNA sequencing (scRNA-Seq) has emerged as a powerful tool for disentangling this complexity [1]. A major challenge in scRNA-Seq of blood is the analysis of granulocytes, in particular neutrophils. These cells are pivotal to immunological responses but are recalcitrant to isolation from blood without inducing activation artifacts, thereby perturbing their transcriptomic integrity [2]. Neutrophils are lost during cryopreservation, making the banking of samples impossible. The difficulty of neutrophil handling for scRNA-Seq necessitates the immediate processing of samples, a workflow incompatible with the logistical demands of large-scale, multi-centre studies.

Innovations in commercial fixed scRNA-Seq methodologies promise to ameliorate the above limitations by preserving RNA integrity post-fixation [3]. Our dataset evaluates two such commercial kits for fixed scRNA-Seq. Our overall goal was to assess the feasibility of distributed collection and centralised processing of fixed whole blood samples.

## 3 Data Description

**Fig. 1:**
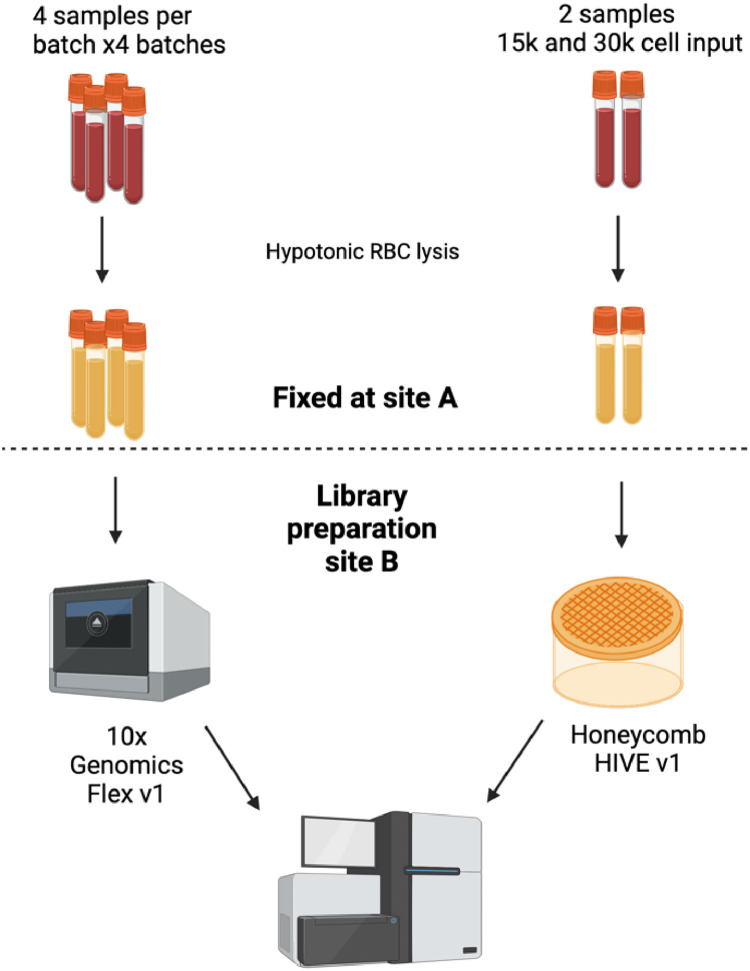
Experimental design. Whole blood samples from healthy donors were depleted of RBCs and fixed at site A. They were shipped to site B for library preparation and sequencing.

The dataset includes 2 technology platforms; Honeycomb HIVE v1 and 10x Genomics Flex v1. This consists of 2 batches of HIVE and 4 batches for 10x Genomics. In total there are 18 samples; 2 for HIVE and 16 for Flex. The underlying samples are red blood cell-depleted whole blood from healthy donors (Fig. 1). For 10x Genomics Flex batch 3, we received a replacement set of library preparation reagents due to poor performance of this batch (“batch 3A”). As instructed by a 10x Genomics field application specialist we performed a second capture reaction of remaining material from batch 3A = “batch 3B”. It is important to note scRNA-Seq platforms and batches were performed on different days.

Files relevant to each sample are raw RNA-seq fastq files and processed cell-by-gene count matrices saved as SingleCellExperiment objects displaying a gene symbol and raw gene count. These files are accessible via GEO. Ensembl gene symbols are represented in rowData. Key metadata relating to the samples include the batch number, which reflects the experimental unit, storage time from fixation to library preparation, and the number of cells fixed and used for library preparation. Key quality control metrics include the total number of molecules detected per cell (library size), the number of genes detected, and the percentage of molecules from mitochondrial genes. A full metadata description is provided in the file “3_examine_metadata,” available at the GitHub repository. The provided GitHub repository also hosts files relevant to preprocessing the data from raw inputs.

## 4 Experimental Design, Materials and Methods

### 4.1 Sample collection

Blood was collected from healthy volunteers through the Skin and Health Institute (Melbourne, Australia). Blood samples were collected into either NaCit or EDTA collection tubes. Sample collection was approved by the Bellberry Human Research Ethics Committee (HREC).

### 4.2 Cell isolation

RBCs were lysed for 15 mins in 1x ACK Buffer (Biolegend Cat no: 420301) protected from light. Cells were washed in PBS twice by centrifugation at room temperature for 5 min at 350g. Cells were counted using the trypan blue exclusion method.

### 4.3 Fixation

Fixation was performed according to each manufacturer-specific protocol. For HIVE the fixative is not disclosed. For 10x Genomics it is 4% paraformaldehyde with an undisclosed enhancer. The number of cells fixed is indicated in the SingleCellExperiment object metadata.

### 4.4 Honeycomb HIVE Library preparation

Cells were counted using trypan blue exclusion and either 30,000 or 15,000 cells were loaded onto the HIVE device as per manufacturer's instructions. Cell-loaded HIVES were stored at -20°C for 3 weeks. Honeycomb HIVE libraries were processed with manufacture's protocol cat 10347 v.0.3 revA.

### 4.5 Genomics Flex v1 Batch 1 and 2

Batches 1 and 2 were performed with Chromium Fixed RNA Profiling Reagent Kits for Multiplexed Samples CG000527, Rev B. Batches 3 and 4 were performed with, Rev D. There are no material differences in reagents or workflow with Rev B being a more detailed protocol than B. The number of cells hybridized to gene-specific probes is indicated in the SingleCellExperiment object metadata.

### 4.6 Sequencing

Samples were sequenced on a NextSeq2000 P3 flow cell targeting 10,000 – 20,000 reads per cell.

### 4.7 Bioinformatics preprocessing

HIVE libraries were preprocessed with Honeycomb BeeNet, v1.1. Due to difficulties in automated cell annotation of neutrophils in scRNA-Seq data, the top 10,000 cell barcodes were selected for downstream analysis. The capture rate of HIVE v1 is 20%, therefore 6,000 and 3,000 cells are expected in the dataset. The human genome reference was GRCh38.104.

**Fig. 2:**
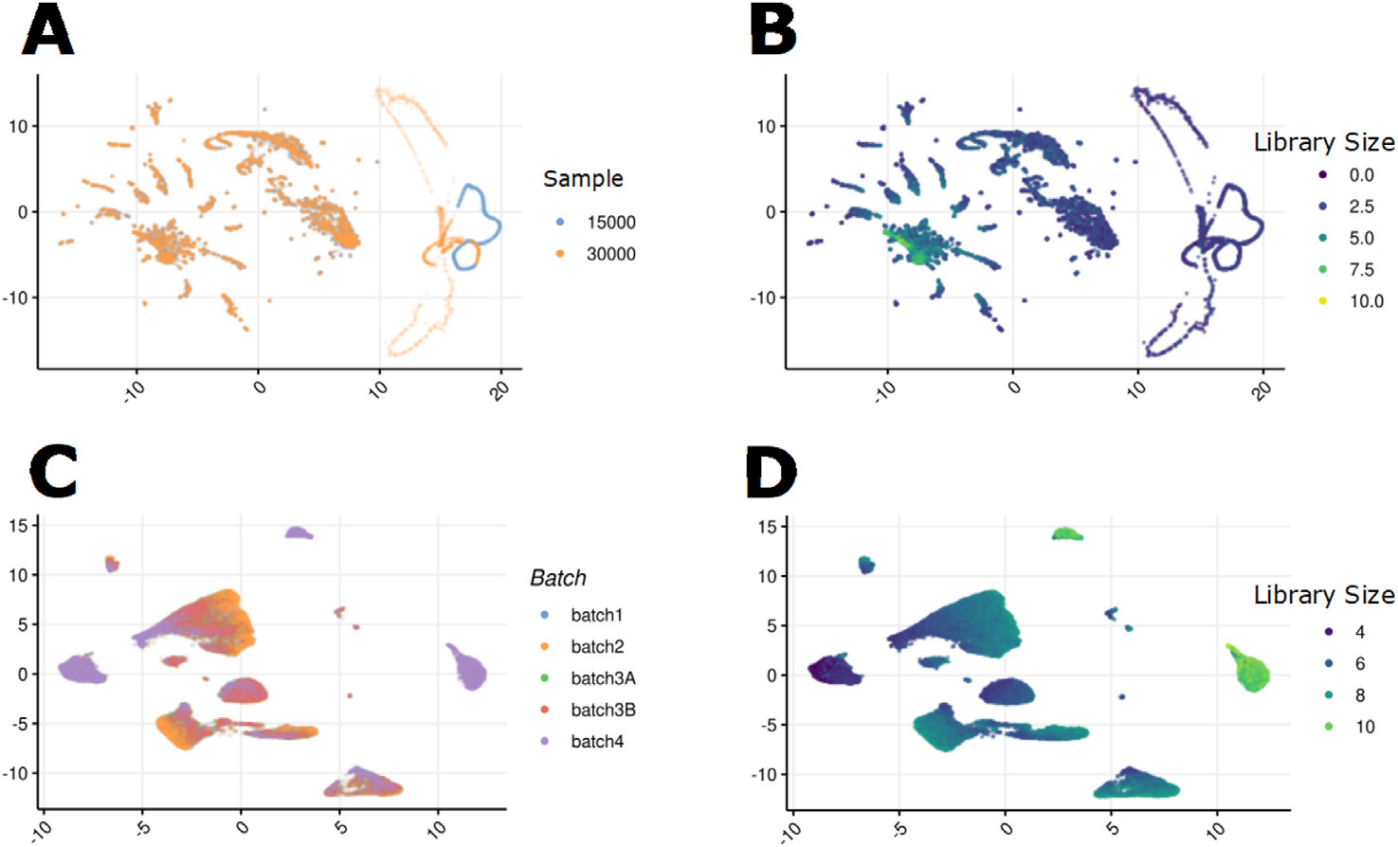
Dimension reduction visualization of data. Cells underwent principal component and uniform manifold approximation and projection prior to visualisation. HIVE cells are coloured for the number of cells input into the capture device (A) and log10 library size in counts per million (B). 10x Genomics cells are coloured for the number of cells input into the capture device (C) and log10 library size in counts per million (D).

10x Genomics Flex libraries were preprocessed with Cell Ranger multi v7.1.0, the –force-cells argument was used to retain the expected number of droplets, based on laboratory cell counts, for downstream analysis (Fig. 2). The probe set reference was v1.0.1 and human genome reference GRCh38-2020-A.

The outputs of these platform specific preprocessing tools were read into R to generate SingleCellExperiment objects [4,5].

## Limitations

Limitations relate to the control of technical effects in our dataset. Although all samples were from healthy RBC-depleted blood, the donor identity, as well as the fixation and processing dates, differed between the Honeycomb HIVE and 10x Genomics Flex. HIVE experiments were conducted before Flex became commercially available. In an attempt to improve the quality of our transcriptomic data we modified the number cells fixed and input to probe hybridisation from batch to batch for 10x Genomics Flex data. This is clearly annotated in the experimental metadata. We did not carefully control the time from sample fixation to library preparation, though we remained within the limits prescribed by 10x Genomics of less than six months. The time from sample fixation to library preparation is also annotated in metadata.

Whole blood was collected at a single site and processed at another distinct single site. Due to this constraint it was not possible to generate fresh whole blood scRNA-Seq data. Only healthy blood donor samples were collected, it is possible certain disease states affect sample fixation and processing performance.

## Ethics Statement

Informed consent was obtained from all individual participants prior to inclusion in the study. The study was performed according to the principles of the 1964 Helsinki declaration. Sample collection was approved by the Bellberry Human Research Ethics Committee (HREC) HREC2012-05-812-A-22.

## CRediT Author Statement

Conceptualization: D.V.B., A.S, M.E.R., A.H., M.A., J.F., R.B.

Methodology: D.V.B., A.S.

Software: D.V.B., Y.Y., Y.Y.

Data curation: D.V.B.

Writing: D.V.B.

Supervision: D.A.Z., P.H., M.E.R., A.H., M.A., J.F., R.B.

Reviewing and Editing: A.S, M.E.R, R.B

## Data Availability

Gene expression OmnibusA Dataset Examining Technical Factors on Fixed Whole Blood Single-Cell RNA-Seq (Original data)
